# *Escherichia coli* Deletion Mutants Illuminate Trade-Offs between Growth Rate and Flux through a Foreign Anabolic Pathway

**DOI:** 10.1371/journal.pone.0088159

**Published:** 2014-02-04

**Authors:** Kelly C. Falls, Aimee L. Williams, Anton V. Bryksin, Ichiro Matsumura

**Affiliations:** Department of Biochemistry, Emory University School of Medicine, Atlanta, Georgia, United States of America; University of Houston, United States of America

## Abstract

Metabolic engineers strive to improve the production yields of microbial fermentations, sometimes by mutating the genomes of production strains. Some mutations are detrimental to the health of the organism, so a quantitative and mechanistic understanding of the trade-offs could inform better designs. We employed the bacterial luciferase operon (*luxABCDE*), which uses ubiquitous energetic cofactors (NADPH, ATP, FMNH_2_, acetyl-CoA) from the host cell, as a proxy for a novel anabolic pathway. The strains in the *Escherichia coli* Keio collection, each of which contains a single deletion of a non-essential gene, represent mutational choices that an engineer might make to optimize fermentation yields. The Keio strains and the parental BW25113 strain were transformed with a *luxABCDE* expression vector. Each transformant was propagated in defined M9 medium at 37°C for 48 hours; the cell density (optical density at 600 nanometers, OD_600_) and luminescence were measured every 30 minutes. The trade-offs were visualized by plotting the maximum growth rate and luminescence/OD_600_ of each transformant across a “production possibility frontier”. Our results show that some loss-of-function mutations enhance growth *in vitro* or light production, but that improvement in one trait generally comes at the expense of the other.

## Introduction

Metabolic engineers seek to optimize the environmentally benign biosynthesis of pharmaceuticals, materials, fuels and other economically valuable compounds [1]. The economic impact of any such project, however, is predicated upon yield. For example, the pathway that produces artemisinin, an anti-malarial drug, in engineered yeast received much well-deserved attention [2]. It would not, however, have been cost-effective without substantial, but less appreciated, strain improvement and fermentation process development [3]. Most economically valuable pathways are anabolic (energy-consuming) and non-essential to the host organism *in vitro*. They utilize intermediates produced by endogenous pathways, including the common energetic cofactors that would otherwise have entered other pathways: adenosine triphosphate (ATP), nicotinamide adenine dinucleotide (NADPH), flavin mononucleotide (reduced form, FMNH_2_), and acetyl-coenzyme A (acetyl-CoA). The metabolic pathways of any cell are interconnected, so it remains difficult to know *a priori* how perturbation of one pathway will affect the flux through a heterologously expressed pathway and the health of the cell.

The experimental tools of functional genomics make it possible to address this question. Transposons can be inserted at nearly random chromosomal positions, thus creating populations of insertion mutants [4], [5]. Alternatively, Mori and his colleagues used homologous recombination to create the Keio collection, which consists of 3985 otherwise isogenic *Escherichia coli* strains, each missing a single open reading frame [6]. Hara *et al*. subsequently propagated each strain in rich medium for 24 hours, permeated the cells via osmotic shock, and reacted the released ATP with firefly luciferase and its substrate luciferin [7]. They measured the optical density (600 nm) of each culture, and its light emission, in a microplate luminometer, and identified numerous deletion mutations that exhibited increased ATP production (per cell over a minute) in the stationary phase. This observation suggests that wild-type *E. coli* cells produce ATP for processes not essential for growth in rich medium (Lysogeny Broth +2.8% glucose), and that the inactivation of these processes by certain deletion mutations can increase the pool of intracellular ATP for artificial anabolic processes. This interpretation, which we call the “free lunch hypothesis,” seems sensible, as the domestication of plants and animals generally produces breeds that are economically more valuable but less fit in the wild.

We wondered whether the free lunch hypothesis could be generalized to all energetic cofactors throughout the bacterial life cycle. We employed the bacterial Lux pathway because it utilizes many of the common exchangeable cofactors (ATP, NADPH, FMNH_2_ and fatty acids produced from acetyl-CoA) and functions within living cells. The bacterial *lux* operon encodes five proteins that form two complexes. The *Lux*CDE complex uses ATP and NADPH to catalyze the reduction of fatty acids into fatty aldehydes. The *Lux*AB luciferase uses FMNH_2_ and oxygen to catalyze the conversion of the fatty aldehyde back into a fatty acid, with the concomitant release of a photon [8]. Here we describe the expression of the *lux* operon within each of the Keio strains, and the measurement of light emission and cell density over 48 hours of growth. We show that the wild-type *E. coli* genome is nearly optimal for growth rate and light production, and that deletion mutations that enhance one trait usually diminish the other.

## Materials and Methods

### Bacterial strains, media and plasmids

The Keio knockout collection [6] and the parent BW25113 *E. coli* strain were obtained from Hirotada Mori (Nara Institute of Science and Technology, Nara, Japan) in March 2006, immediately after they became available; our stocks have not since been propagated. The Lysogeny Broth (or Luria-Bertani, LB) was from EMD, and the M9 medium and bacto-agar were from Difco. Restriction enzymes, T4 DNA ligase, Phusion DNA polymerase, and the 1 kb ladder were from New England Biolabs (Ipswich, MA). DNA oligonucleotides were from IDT (Coralville, IA), and custom DNA sequencing services were provided by Macrogen (Rockville, MD). Polymerase chain reaction (PCR)-grade deoxynucleotide triphosphate (dNTP) kits were from Roche Applied Science (Indianapolis, IN).

The polyethylene glycol (MW = 8,000) was from Fluka (now a division of Sigma-Aldrich, *vide infra*). DNA was purified with QIAprep Gel Extraction and Spin Miniprep kits (Qiagen, Valencia, CA). Isopropyl-β-D-thio-galactoside (IPTG) was from Gold Biotechnology (St. Louis, MO). All other chemicals were from Sigma-Aldrich (St. Louis, MO). The microtiter plates were from Nunc (96-well, clear, flat bottom) or Corning (384-well, white or white with clear bottom). The construction of the pIMBB-T5-*lux* expression vector was previously described [9]. Briefly, a series of *lux* ORFs (*luxA, luxB, luxC, luxD* and *luxE*) was PCR amplified from the *Photorhabdus luminescens* (ATCC 29999) chromosome and separately cloned into pIMBB. The internal Xba I site within *luxD* was eliminated by site-directed mutagenesis. Each BioBrick was combined with an optimized ribosomal binding site (rbs). The rbs-ORF BioBricks were combined with each other, the T5-promoter, and two *lac* operators to form pIMBB-T5-*lux*.

### High Throughput transformation of KEIO collection with pIMBB-T5-Lux

The modified polyethylene glycol/dimethyl sulfoxide (PEG/DMSO) protocol, originally developed by Chung *et al.* [10], was used to transform each of the Keio strains with the pIMBB-T5-*Lux* plasmid. This method, unlike others, doesn't require the preparation of competent cells beforehand and can take as little as 5–6 hours per batch. The Keio strains were delivered in 96-well plates. Each was seeded with a 96-pin microplate replicator into flat bottom 96-well plates (Nunc); each well contained 20 microliters of fresh LB supplemented with 10 mM MgSO4 and 50 mM 2-(N-morpholino)ethanesulfonic buffer (pH 6.1). The microtiter plates were agitated at 600 rpm in an ATR Microtitertron shaker until the cells were in the exponential phase (OD_600_ = 0.4–0.7). Growth temperature (17 to 37°C) did not affect transformation efficiency.

A Thermo Scientific Multidrop 384 coupled to a Titertek Titan plate stacker was used to add 20 microliters of 2X TSS (2X LB, 50 mM MgCl2, 50 mM MgSO4, 20% PEG 8000, 10% DMSO) containing pIMBB-T5-*Lux* at a concentration of 1 ng/microliter to each micro-culture. Plates were shaken briefly for 2 minutes at 600 rpm and incubated on ice for 30–60 minutes. The Multidrop 384 dispenser was used to add 200 microliters of LB to each microculture. The microplates were transferred to the ATR Microtitertron, and shaken at 33°C for 1 hr at 600 rpm to allow expression of the ampicillin (Amp)-resistance gene. The dispenser was used to add 10 microliters of ampicillin stock solution (3.5 mg/mL) to each well (final concentration of 140 micrograms/mL. The microcultures were replicated using a 96-pin microplate replicator into new plates; each well contained 200 microliters of fresh LB supplemented with either Amp (100 micrograms/mL), for BW25113 strain, or Amp and kanamycin (Kan, 50 micrograms/mL), for Keio mutants. The *E. coli* cells were transformed in 96 well microtiter plates, so the resulting transformants were arrayed in the same order and configuration as the original (untransformed) Keio collection [6]. The *E. coli* micro-cultures were allowed to grow to saturation overnight at 33°C and 600 rpm. Saturated cultures were supplemented with glycerol (final concentration of 10%), shaken for 2 minutes at 600 rpm, frozen and stored at −80°C.

The transformants were propagated to saturation in liquid LB supplemented with ampicillin (and kanamycin for the Keio strains), then reformatted in 384-well microtiter plates; the *lux*/BW25113 was replicated in the wells of a 384-well microtiter plate while the 3747 *lux*/Keio strains were distributed among 12×384-well plates. The micro-cultures were propagated overnight at 30°C, and subsequently frozen at -80°C. PCR using primers designed to recognize the kanamycin phosphotransferase gene (used to knock out genes), and those specific for adjacent regions, were used to confirm the identities of arbitrarily chosen transformed Keio strains in each of the 12 microtiter plates (data not shown).

### Luminescence and Growth Assays

Frozen, transformed Keio strains stored in 384-well plates were thawed out and diluted about 50-fold with a 384-pin replicator into new plates; each well contained 50 microliters of M9 supplemented with 1 mM thiamine, 0.4% glucose, 250 micromolar isopropyl-β-D-thiogalactoside (IPTG), and 100 micrograms/mL ampicillin (no kanamycin). The kanamycin resistance marker in the Keio strains does not affect cell growth in the absence of antibiotic, as knock-outs of single copies of multicopy genes result in wild-type-like strains (data from 42 such Keio strains not shown). Each microtiter plate was sampled three times on different days, and each of the recipient plates were separately assayed with a BioTek Synergy2 microplate reader. OD_600_ and luminescence were measured at 30 minute intervals for 48 hours. Plates were shaken continuously at medium speed, and temperature was kept at 37°C. Absorbance was read at 600 nm. Luminescence was recorded at the following settings: 1.0 sec integration time, a 4.5 mm read height, and a 130 gain.

### Data Analysis

Data from the BioTek Synergy2 microplate reader was acquired and analyzed with the Gen5 software, then exported to Excel files (raw data available upon request). The derived values, namely maximum growth rate (mOD_600_/min), maximum optical density, maximum luminescence, integrated OD_600_ and integrated luminescence, from the three technical replicates of each *lux*/Keio plate were manually combined into one Excel document per plate. Average values and standard error were calculated in Microsoft Excel, and the resulting parameters derived from the entire Keio collection were consolidated in a single Excel document (Table S1). Data from three technical replicates of the *lux*/BW25113 plate were similarly combined in a separate document (Table S2). Kaleidagraph 3.5 (Synergy Software) was used to create the figures.

Liquids in the outermost wells of 384-well microtiter plates tend to evaporate more quickly than those situated in the interior; bacterial cultures at the edges increase in cell density up to 20% faster than those in the middle. Such edge effects are well-documented [11], [12], commonplace and difficult to avoid. To demonstrate the latter, the parental control strain (*lux*/BW25113) was propagated in 384 well microtiter plates with lids containing standard media (50 microliters M9-ampicillin) in a humidity-controlled ATR Microtitertron (600 rpm at 80% humidity, 33°C for 23 hours). The OD_600_ was manually measured in a SpectraMax M5 plate reader (Molecular Devices) at 5, 8 and 23 hours; edge effects similar to those recorded during growth in the Biotek Synergy2 were observed. We therefore calculated the average maximum growth rate (mOD_600_/min) values of cultures in each of the eight loops of wells, from the outermost (A1-24, P1-24, 1A-P, 24A-P) to the innermost (H8-17, I8-17) during continuous growth in the Synergy2. The values derived from the outer three loops were on average 1.35, 1.16 and 1.05-fold greater, respectively, than those of the inner five loops. The maximum growth rate values of all cultures (lux/BW25113 and lux/Keio) in the outer three wells were corrected by multiplying them by 0.74, 0.86 and 0.95 respectively. Some mutants probably respond differently than the parental control strain to reductions in culture volume, but we reasoned that most did not.

## Results

### The productivity of the bacterial luciferase pathway is growth phase dependent

We attempted to transform the 3985 Keio strains (in 96-well microtiter plates) and the parental BW25113 *E. coli* strain with our plasmid-borne *luxABCDE* operon. Approximately 6% (238/3985) of the Keio strains failed to transform after repeated attempts with our high throughput protocol (Table S3). A micro-aliquot of each culture, *lux*/BW25113 and *lux*/Keio, was transferred with a 384-pin replicator into microtiter plates containing fresh minimal medium supplemented with ampicillin to select against non-transformants and isopropyl-β-D-thiogalactoside (IPTG) to induce the expression of the *luxABCDE* operon. Each plate was shaken at 37°C for 48 hours in a Synergy2 microplate spectrophotometer/luminometer; the optical density (600 nm) and light emission of each micro-culture were measured every 30 minutes. Three technical replicates were made from each stock micro-culture, so the parental *lux*/BW25113 strain was propagated and monitored 1152 times (3×384).

**Figure 1:**
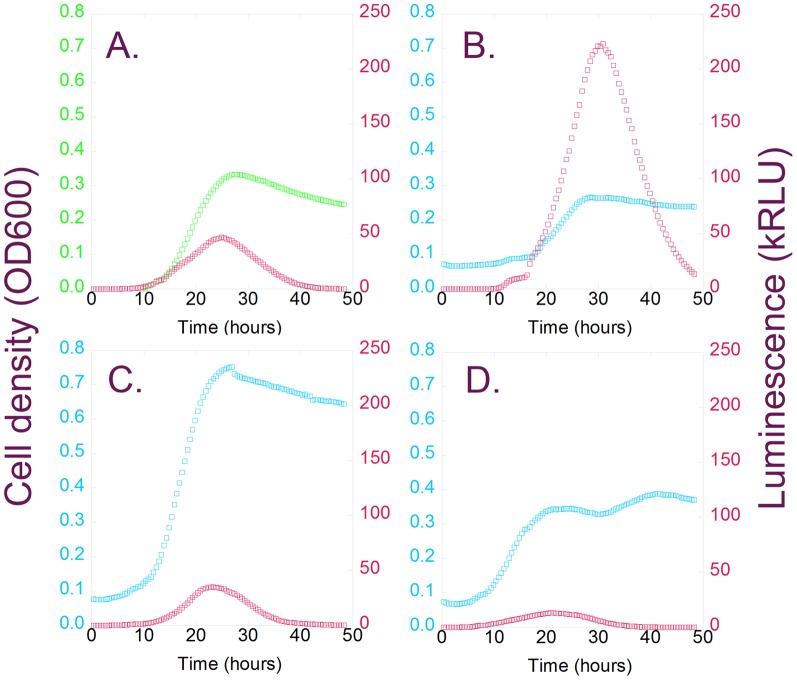
Deletion mutations can affect the growth of *Escherichia coli*, and the production of light from bacterial luciferase. *E. coli* Keio strains, and the parental BW25113 strain, were transformed with a bacterial luciferase expression vector (pIMBB-T5-*lux*). The transformants were shaken in minimal medium at 37°C for 48 hours; the optical density at 600 nm (green or blue) and luminescence (red) were recorded every 30 minutes. Data from the (A) parental *lux*/BW25113 control, and three representative Keio transformants, (B) *lux*/Δ*ybhC*, (C) *lux*/Δ*lon* and (D) *lux*/Δ*fiu*, are presented here. The growth curves of the three Keio strains differ from that of the BW25113 control, but the luminescence curves are all similar in shape.

Light production rose as cell density increased, suggesting that the observed lag times were a function of cell physiology rather than the sensitivity of the spectrophotometer. Luminescence peaked as the cells entered stationary phase (Figure 1a), which suggests that the intracellular concentration of at least one energetic cofactor decreases as the cells left log phase, presumably due to nutrient depletion. The mutant cultures exhibit the same patterns of light production, although their growth patterns vary significantly with regard to lag time, maximum growth velocity, and cell death rate (Figure 1b–c). Some mutants exhibit two phase growth, with light production diminishing after the first phase (Figure 1d); we hypothesize that the second phase begins as growth on exported acetate commences [13], thus leading to decreased light production.

### Some lux/Keio strains exhibit faster growth, while others produce more light

**Figure 2:**
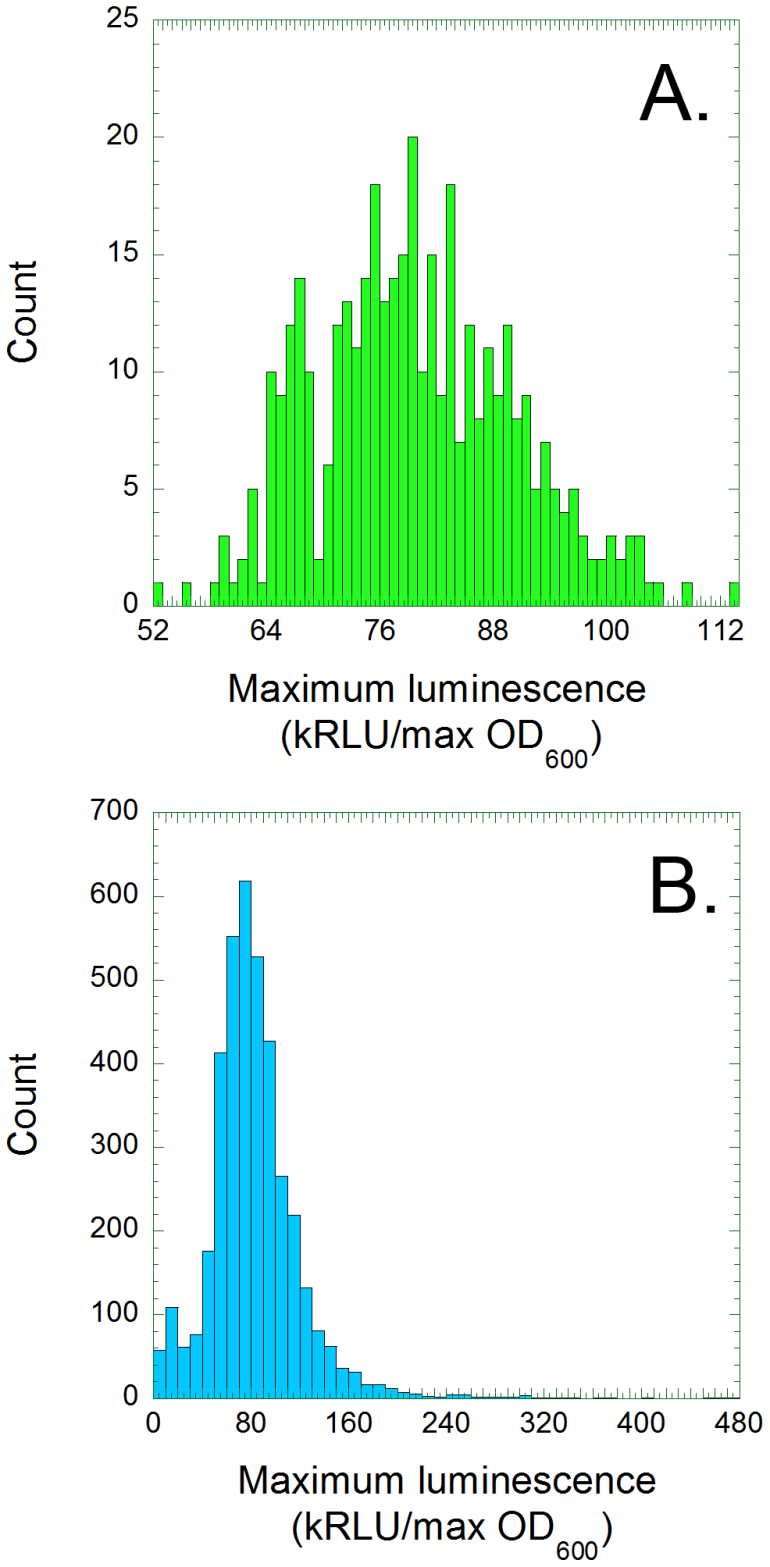
Light production per cell is normally distributed among the 384 *lux/BW25113 parental control replicates (A), but not among the 3747 (N = 3) mutant lux/Keio cultures (B).* The average maximum luminescence (Relative Light Units) of each transformant was divided by its maximum OD_600_, and the resulting values were plotted on histograms.

**Figure 3:**
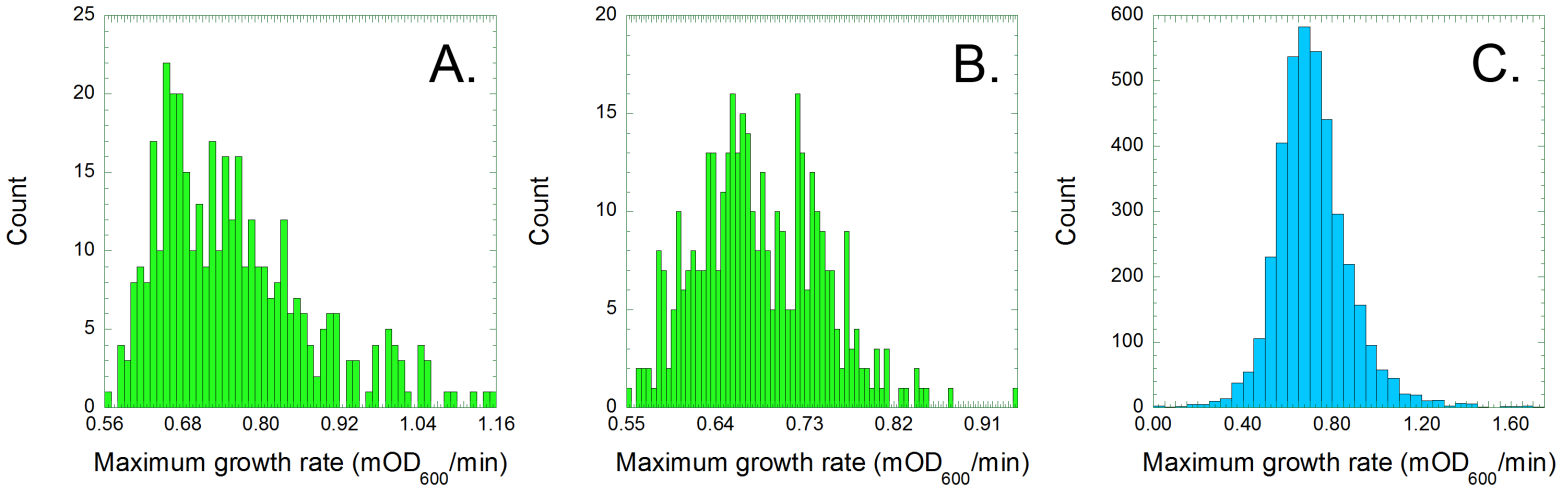
Maximum growth rates of 384 *lux*/BW25113 parental control replicates (A) are normally distributed when corrected for edge effects (B). The corrected maximum growth rates of the 3747 (N = 3) mutant *lux*/Keio cultures (C) are distributed more widely than would a control population of the same size.

We sought to test the free lunch hypothesis, and to understand the biochemical nature of the tradeoffs between anabolism and cellular health, so we focused our analysis upon two parameters that were derived from our data. We identified the maximum luminescence (Relative Light Units) and maximum optical density (OD_600_) of each culture. Both values were recorded in every experiment as the cells reached the end of logarithmic growth, so maximum luminescence divided by maximum optical density (lum/OD_600_) reflects the rate at which cells produce light (Figure 2). We also derived the maximum growth rate (mOD_600_/minute), which reflects the rate at which cells produce other cells (Figure 3). The mean and standard error values of both parameters were calculated for each transformed mutant (N = 3). The maximum growth rate values were corrected (Materials and Methods) to compensate for increased evaporation from the wells at the edges of each plate.

The average lum/OD_600_ values of the 384 replicates of *lux*/BW25113 are normally distributed (Shapiro Wilks test statistic >0.99, P-value = 0.03). The values range from about 52,400 to 113,000 RLU/OD_600_, with an average of 79,800 and a standard deviation of 10,400 (Figure 2a). The comparable lum/OD_600_ values of the 3747 *lux*/Keio transformants distributed more broadly and asymmetrically (Shapiro Wilk test statistic = 0.86), ranging from 2,820 to 479,000 RLU/OD_600_, with an average of 82,900 and a standard deviation of 39,100 (Figure 2b). A normally distributed population of 3747 *lux*/BW25113 replicates would be expected to vary from 43,800 to 116,000, but 357 *lux*/Keio transformants (9.5%) fell below that range while 524 (14%) were above.

The corrected maximum growth rate values of the 384 *lux*/BW25113 replicates are also normally distributed (Shapiro Wilk test statistic = 0.98, P-value = 0.0002); the values range from 0.55 to 0.94 mOD_600_/min, with a mean of 0.68 and a standard deviation of 0.063 (Figure 3b). Again, the mutants exhibited greater variation than did the parental controls (Kolmogorov–Smirnov maximum D = 0.230, p<0.0000001). The comparable maximum growth rate values of the *lux*/Keio strains ranged from 0.01 to 1.75, with a mean of 0.71 and a standard deviation of 0.17 (Figure 3c). A population of 3747 *lux*/BW25113 replicates should exhibit a range of maximum velocities from 0.46 to 0.90 mOD_600_/min (presuming a null hypothesis in which the mutants were wild-type-like), but 148 *lux*/Keio transformants (3.9%) grew more slowly than expected while 450 (12%) grew more quickly. Previous workers did not observe any *E. coli* strains with enhanced fitness among a collection of 226 transposon insertion mutants [14], but the growth of their cells was not hampered by a constitutive expression vector.

**Figure 4:**
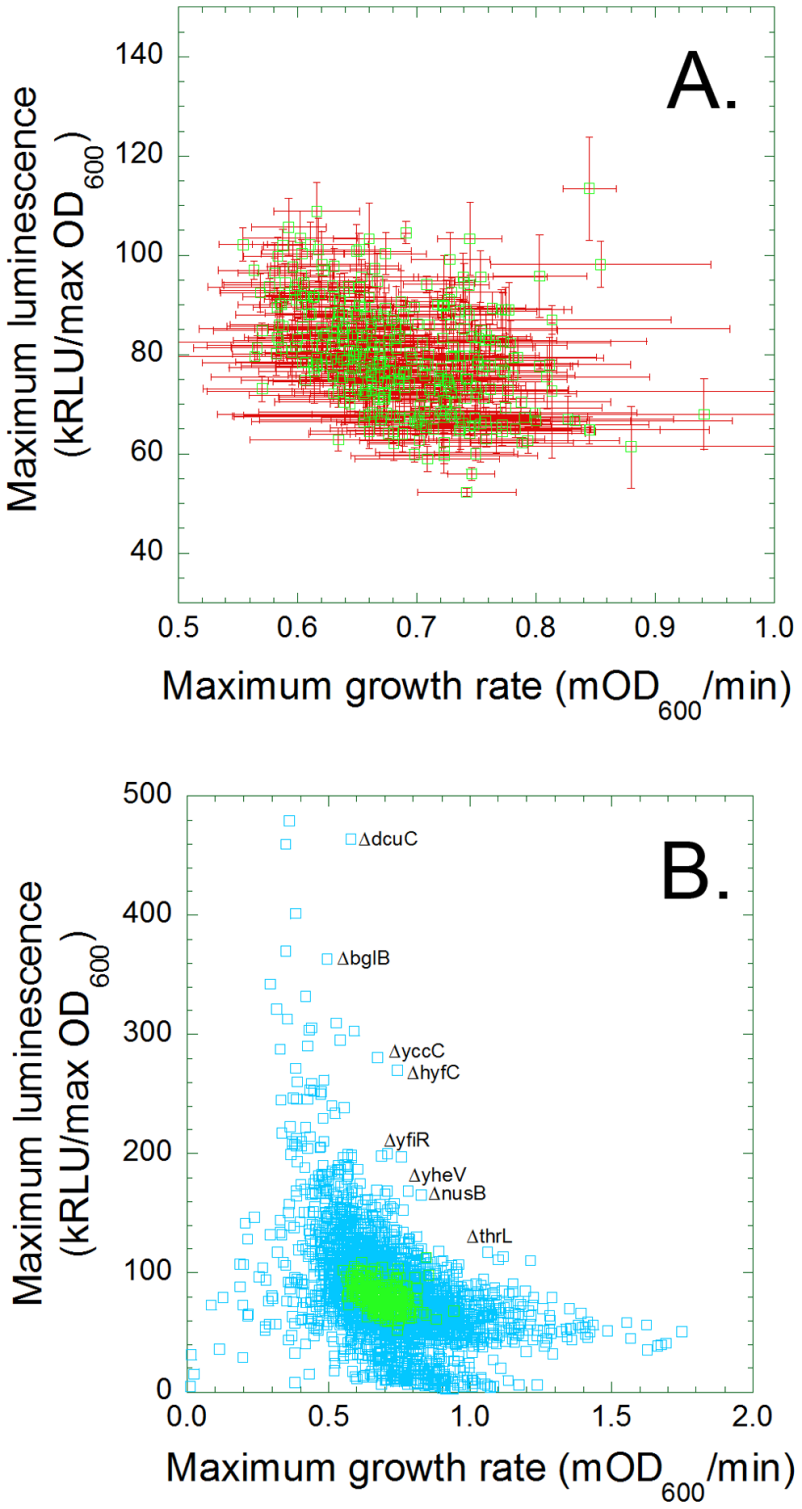
The maximum growth rate and luminescence per cell of 384 lux/BW25113 control replicates (A) or *lux*/Keio transformants (B) are shown in scatter plots. The distances between points (green squares, A) reflect the variations among isogenic *lux*/BW25113 cultures in a 384 well plate; the error bars represent the variations between repetitions of the experiment (N = 3). The same data (green squares, B) was plotted among those of the *lux*/Keio transformant (blue squares, B) to facilitate direct comparisons.

The fitness and luminescence values associated with 3747 *lux*/Keio strains can be plotted on a two dimensional trade-off curve, with maximum growth rate (mOD_600_/min) on the X-axis and maximum luminescence/maximum optical density on the Y-axis (Figure 4). Economists use a similar plot, the “production possibility frontier” (PPF), to depict the quantities of two commodities that could be manufactured by an organization (or a country) within a given interval of time from a fixed amount of the factors of production. Our PPF plot (Figure 4) shows that most *lux*/Keio strains are similar to the parental control (*lux*/BW25113) in growth rate and light production. Many others grow more slowly and/or produce less light than the control. A few either produce more light than the ancestor strain but these tend to grow more slowly, or grow quickly but produce less light. We investigated some clones at the margins of the frontier to see whether the *E. coli* offers any free lunches.

### A few lux/Keio transformants outshine the parental lux/BW25113 ancestor

Metabolic engineers are generally more concerned with fermentation yields than with growth rates. Chromosomal mutations that increase the productivity of a foreign anabolic pathway, represented in this case by luminescence/OD_600_, could thus be economically valuable [4]. Such mutations, however, could not be combined to further improve yields unless they were neutral in net effect upon cellular health. Several lux/Keio transformants exhibited statistically significant improvements over the lux/BW25113 control in light production with only modest effects upon growth rate: Δ*hyfC*, Δ*yccC*, Δ*dcuC*, Δ*bglG*, Δ*yfiR*, Δ*yheV*, Δ*nusB*, and Δ*thrL*. Colonies (biological replicates, N = 6) were separately propagated in 300 microliter cultures in M9-ampicillin, then diluted 1∶50 in the same medium, supplemented with 250 micromolar IPTG, in 96 well microtiter plates. The plates were agitated (medium) in our spectrophotometer/luminometer (Biotek Synergy 2) for 24 hours; the optical density (600 nm) and luminescence (Relative Light Units) were measured every 15 minutes.

**Figure 5:**
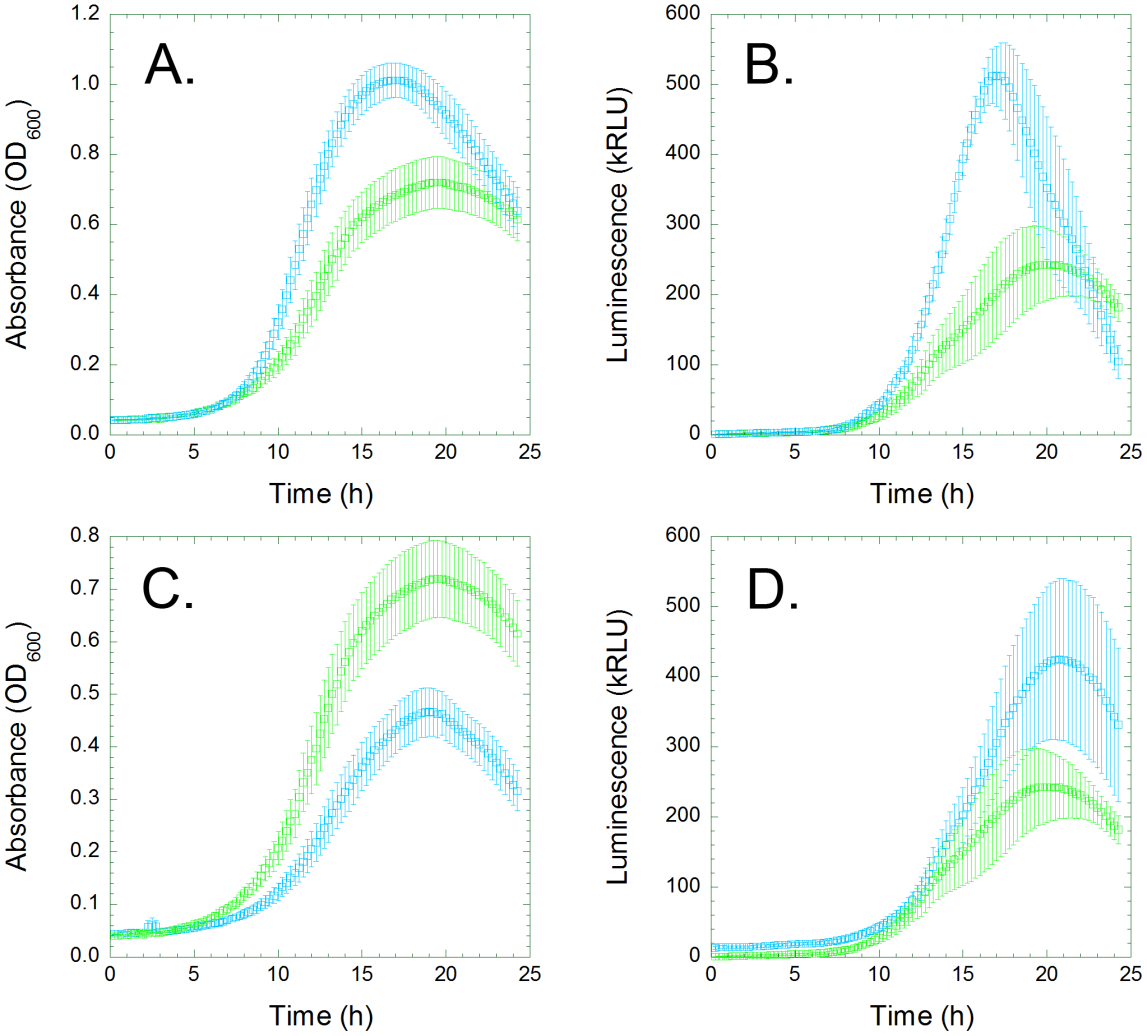
The *lux*/Δ*thrL* and *lux*/Δ*hyfC* strains exhibit improvements over the parental *lux*/BW25113. Each strain was propagated (N = 6) for 24 hours in M9 medium supplemented with ampicillin and IPTG in a Biotek Synergy2 plate reader (37°C, medium shaking); the OD_600_ and luminescence were recorded from each culture every 15 minutes. The lux/Δ*thrL* strain (blue squares, A) grows more quickly and to higher cell density than does the lux/BW25113 (green squares), and produces more light (B). The lux/Δ*hyfC* transformant grows more slowly the parental control (C), but produces more light (D).

Only one of the clones we re-tested, *lux*/Δ*thrL*, exhibited significant improvement over the parental lux/BW25113, in both cell growth (Figure 5a) and light production (Figure 5b). One other, *lux*/Δ*yveH*, exhibited enhanced light production without significant reduction in growth (data not shown). It may be possible to combine these “free lunch” mutations to produce a strain that evinced even better performance, at least under laboratory conditions. Three other transformants, *lux*/Δ*hyfC* (Figure 5c–d), *lux*/Δ*yfiR* and *lux*/Δ*nusB* produced more light at the expense of growth. Three others, lux/Δ*yccC*, *lux*/Δ*dcuC* and *lux*/Δ*blgG*, produced less light than the control lux/BW25113 transformant (data not shown), indicative of the observed imprecision in the initial screen (Figure 4a).

### Different assays produce different results

Hara *et al.* mixed ATP from Keio strains in the stationary phase with firefly luciferase and its luciferin substrate. They reported the “cellular ATP synthetic activity,” the quantity of light relative to the wild-type value divided by cell density (also relative to the wild-type value) of each strain. They identified 21 “*inc*” strains that exhibited >200% relative activity (per cell, compared to the wild-type) and 20 “*dec*” that exhibited <50% relative activity [7]. We expected *a priori* that these strains would have similar phenotypes in our assay, since the *lux*ABCDE pathway is partially dependent on ATP. Contrary to expectation, none exhibited relative activity (maximum luminescence divided by maximum OD_600_) over double that of the *lux*/BW25113 control in our assay (although 112 other strains in our assay exceeded this criterion). Similarly, only seven of the 20 *dec* mutants exhibited less than half of the relative activity of the control (319 other strains fell below this threshold in our assay); nine of the *dec* mutants actually exhibited higher relative activity than the control. The disparity is not surprising. The bacterial luciferase used in our study utilizes several cofactors (ATP, NADPH, FMNH_2_ and fatty acids), and was mostly active at log phase, while the firefly luciferase employed by Hara *et al.* required only ATP (as the luciferase was exogenously added) and was assayed at stationary phase.

Baptist et al. recently cloned the *lux* operon downstream of the acetyl-CoA synthetase (*acs*) promoter. They transformed the Keio strains with their *lux* expression vector and used a colony-based screen to identify *E. coli* genes that normally regulate *acs*. The genes they identified were naturally quite different than our strongest effectors (*vide supra*). The *lux* operon was constitutively expressed in our experiment, so it could be regarded as a negative control for their study and others like it. Alternatively, well-characterized reporter genes, such as *lacZ* or *gfp*, are less sensitive to the metabolic state of the cell and would therefore be more appropriate for genome-wide screens for modifiers of any particular gene of interest.

## Discussion

We sought to test the “free lunch hypothesis,” the notion that the inactivation of anabolic processes that are not essential *in vitro* will increase the energy available to competing processes. Our observations suggest that a few such “free lunch” mutations exist (Figure 5ab), and that the vast majority of mutations that enhance light production also exhibit below average growth rate (Figure 5cd). We conclude that light production competes with the production of new cells. The shape of the PPF informs economists about the opportunity costs of producing each commodity. A straight line reflects constant opportunity costs, which means that resources are not specialized for the production of either output. An inward curving PPF such as ours (Figure 4b) reflects economies of scale, which means that the opportunity cost of manufacturing one output falls as more of it is produced. Our plot suggests to us that the mutations with the largest phenotypic effects tend to be pleiotropic, which means that they disrupt multiple pathways that normally affect growth and light production in opposite ways.

ATP flux does not determine the growth rate of *E. coli*, and ATP yield does not determine cell density at stationary phase [15]. The trade-offs between fitness and light production must therefore be indirect in nature. Genome-scale metabolic models could be used to formulate some mechanistic hypotheses. The *thrL* gene is a negative regulator of the threonine operon [16], so the deletion likely increases the production of this amino acid in minimal medium. YccC, or Etk, is a membrane tyrosine autokinase that participates in a form of polysaccharide secretion [17] not essential in the laboratory. HyfC is a component of hydrogenase 4, which converts formate into CO_2_ and H_2_ [18]. Even the most comprehensive models [19], [20] remain incomplete. The primary functions of one quarter of the genes in *E. coli* K-12, including *yveH and yfiR*, remain unknown [21]. Fundamental biochemical parameters (including the concentrations, rate constants, Michaelis constants of hundreds of catalysts and metabolites) and regulatory properties of many well annotated genes have yet to be experimentally determined. We therefore hope that our data set (Table S2), and others like it, will help others to refine their genome-scale models.

Our study can also inform efforts to improve the biosynthetic yields from genetically modified microorganisms. Engineers are taught to formulate quantitative models, but this generally useful skill can lead some to oversimplify. Some synthetic biologists long for a free-living organism with a minimal genome [22], but we showed here that the deletions of non-essential genes can impart precipitous decreases in biosynthetic yield. *Photorhabdus luminescens*, the source of our *lux* genes, was apparently optimized by evolution to produce light, as it outshines our best deletion mutants (data not shown). It expresses additional specialized genes, such as *luxG* [23], that cannot be phenocopied by loss-of-function mutations. *E. coli* can be engineered to produce 1,3 propanediol at 82% theoretical yield [24], but other transformable species will likely prove to be better starting points for the production of more challenging target compounds.

## Supporting Information

Table S1 Lux Keio parameters. The *lux*/Keio strains were propagated three times in 384 well microtiter plates, the luminescence and optical density at 600 nm were measured every 30 minutes, and the maximum growth rate (max V OD), integrated optical density (area under growth curve, int OD), integrated luminescence (area under luminescence curve, int lum), maximum optical density at 600 nm (max OD), maximum luminescence (max lum) were calculated for each technical replicate.
(XLSX)

Table S2 Lux BW25113 parameters. The parental *lux*/BW25113 control strain was used to seed the wells of a 384 well microtiter plate. Each of the micro-cultures was propagated three times, the luminescence and optical density at 600 nm were measured every 30 minutes, and the growth and light emission parameters (see Table S1 Legend) were calculated.
(XLSX)

Table S3 Unsuccessful transformation. The Keio strains that we were unable to transform with the pIMBB-T5-*lux* expression vector are listed.
(XLSX)
